# The Designed Pore-Forming Antimicrobial Peptide C14R Combines Excellent Activity against the Major Opportunistic Human Pathogen *Pseudomonas aeruginosa* with Low Cytotoxicity

**DOI:** 10.3390/ph17010083

**Published:** 2024-01-09

**Authors:** Vanessa Mildenberger, Daniel Alpízar-Pedraza, Ernesto M. Martell-Huguet, Markus Krämer, Grigory Bolotnikov, Anselmo J. Otero-Gonzalez, Tanja Weil, Armando Rodriguez-Alfonso, Nico Preising, Ludger Ständker, Verena Vogel, Barbara Spellerberg, Ann-Kathrin Kissmann, Frank Rosenau

**Affiliations:** 1 Institute of Pharmaceutical Biotechnology, Ulm University, Albert-Einstein-Allee 11, 89081 Ulm, Germany; vanessa.mildenberger@uni-ulm.de (V.M.); markus-1.kraemer@uni-ulm.de (M.K.); grigory.bolotnikov@uni-ulm.de (G.B.); 2 Center for Pharmaceutical Research and Development (CIDEM), 26th Avenue, No. 1605, Nuevo Vedado, La Habana 10400, Cuba; dalpizarp@gmail.com; 3 Center for Protein Studies, Faculty of Biology, University of Havana, 25 and I, La Habana 10400, Cuba; nestmartell@gmail.com (E.M.M.-H.); aotero@fbio.uh.cu (A.J.O.-G.); 4 Max Planck Institute for Polymer Research Mainz, Ackermannweg 10, 55128 Mainz, Germany; weil@mpip-mpg.mainz.de; 5 Core Facility for Functional Peptidomics (CFP), Faculty of Medicine, Ulm University, 89081 Ulm, Germany; armando.rodriguez-alfonso@uni-ulm.de (A.R.-A.); nico.preising@uni-ulm.de (N.P.); ludger.staendker@uni-ulm.de (L.S.); 6 Core Unit of Mass Spectrometry and Proteomics, Faculty of Medicine, Ulm University, 89081 Ulm, Germany; 7 Institute of Medical Microbiology and Hygiene, University Clinic of Ulm, TBC1 Forschung, Albert-Einstein-Allee 11, 89081 Ulm, Germanybarbara.spellerberg@uniklinik-ulm.de (B.S.)

**Keywords:** antimicrobial peptides, clinical isolates, pore-formation, *Pseudomonas aeruginosa*

## Abstract

The diminishing portfolio of mankind’s available antibiotics urges science to develop novel potent drugs. Here, we present a peptide fitting the typical blueprint of amphipathic and membrane-active antimicrobial peptides, denominated C14R. This 2 kDa peptide consists of 16 amino acid residues, with seven being either hydrophobic, aromatic, or non-polar, and nine being polar or positively charged, strictly separated on opposite sides of the predicted α-helix. The affinity of the peptide C14R to *P. aeruginosa* membranes and its intrinsic tendency to productively insert into membranes of such composition were analyzed by dynamic simulations. Its biological impact on the viability of two different *P. aeruginosa* reference strains was demonstrated by determining the minimal inhibitory concentrations (MICs), which were found to be in the range of 10–15 µg/mL. C14R’s pore-forming capability was verified in a permeabilization assay based on the peptide-triggered uptake of fluorescent dyes into the bacterial cells. Finally, the peptide was used in radial diffusion assays, which are commonly used for susceptibility testing of antimicrobial peptides in clinical microbiology. In comparison to reference strains, six clinical *P. aeruginosa* isolates were clearly affected, thereby paving the way for further in-depth analyses of C14R as a promising new AMP drug in the future.

## 1. Introduction

**Figure 1:**
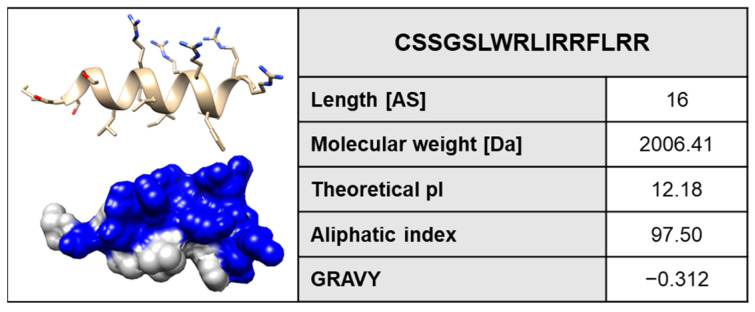
The 3D-structure of the peptide C14R (NH_2_-CSSGSLWRLIRRFLRR) obtained by an ab initio method using AlphaFold2 from Google Colabs (https://colab.research.google.com/github/sokrypton/ColabFold/). Properties of C14R calculated with ExPASy ProtParam. Given are the amino acid sequence, the length, theoretical isoelectric point (pI), the aliphatic index, and the grand average hydropathy index (GRAVY).

In 2017, the WHO had already classified carbapenem-resistant strains of the Gram-negative opportunistic pathogenic bacterium *Pseudomonas aeruginosa*, along with *Acinetobacter baumannii* and *Enterobacteriaceae*, as one of the most threatening pathogens for which the greatest need of action exists to develop new powerful antibiotics [1]. A promising class of drug molecules with a wide range of activity against viruses, bacteria, fungi, and parasites are antimicrobial peptides (AMPs), occurring in nature in an impressive variety of effective molecules [2,3,4,5]. More than 3100 AMPs with certain therapeutic potential have been reported in recent decades [6], which is of particular interest in the steadily progressing age of resistance development [7]. They can be isolated from different organismic resources, with mollusks representing a prominent phylum in which interesting molecules have been identified and further developed [8,9,10,11,12,13,14]. Classical AMPs are characterized by a simple physicochemical mode-of-action due to the way that they can assemble into pores of the target biomembrane via interactions through the peptide residue sidechains [15,16]. A structural motif typical for such peptides is a distinctive separation of hydrophilic and hydrophobic residues forming a pronounced amphipathic architecture on opposite sides of the peptide molecule [17,18,19,20]. Several approaches have been introduced and successfully been used to identify, optimize [21], or ab initio design new functional sequences (nicely reviewed in Torres et al., 2019 [22]). Based on studies involving complete substitution libraries of small peptides against *P. aeruginosa* with sequence lengths around twelve amino acids [23], and based on findings suggesting that tryptophan and arginine (R and W) residues generally represent important improvements in the membrane activity of peptides [24], BP100 and its derivatives peptides originating from another synthetic library were isolated and sequence optimized accordingly [25,26]. BP100 as a “lead structure” was active against Gram-negative bacteria per se, with drastically reduced activity against Gram-positive bacteria, whereas R-BP100 and RW-BP100 had significantly improved activities against Gram-positive and also *P. aeruginosa* [25]. In accordance with these peptides, we here present in detail the anti-*P. aeruginosa* and pore-forming activity of another RW-rich peptide molecule of this type, which was designed to meet the requirements of the amphipathic antimicrobial peptide blueprint, which we tentatively have named C14R (Figure 1). This 2 kDa peptide consists of 16 amino acids, with seven being either hydrophobic, aromatic, or non-polar, and nine being polar or positively charged, strictly separated on opposite sides of the predicted α-helix (Figure 1). The affinity of the peptide C14R to *P. aeruginosa* membranes and its intrinsic tendency to productively insert into membranes of such composition was analyzed by dynamic simulations. Its biological impact on the viability of two different *P. aeruginosa* reference strains was demonstrated by determining the minimal inhibitory concentrations (MICs), which were found to be in the range of 10–15 µg/mL. C14R’s pore-forming capability was verified in a permeabilization assay based on the peptide-triggered uptake of fluorescent dyes into the bacterial cells. Finally, the peptide was used in radial diffusion assays, confirming a pronounced antibacterial activity of C14R against six clinical *P. aeruginosa* isolates, comparable to the activity observed against reference strains. With an overall absent cytotoxicity and no hemolytic activity, C14R especially qualifies as a possible treatment option due to its low or possibly no in vivo toxicity and can be expected to be used in systemic application. These promising results warrant further in-depth analyses of C14R as a candidate for developing novel future AMP drugs.

## 2. Results

**Figure 2:**
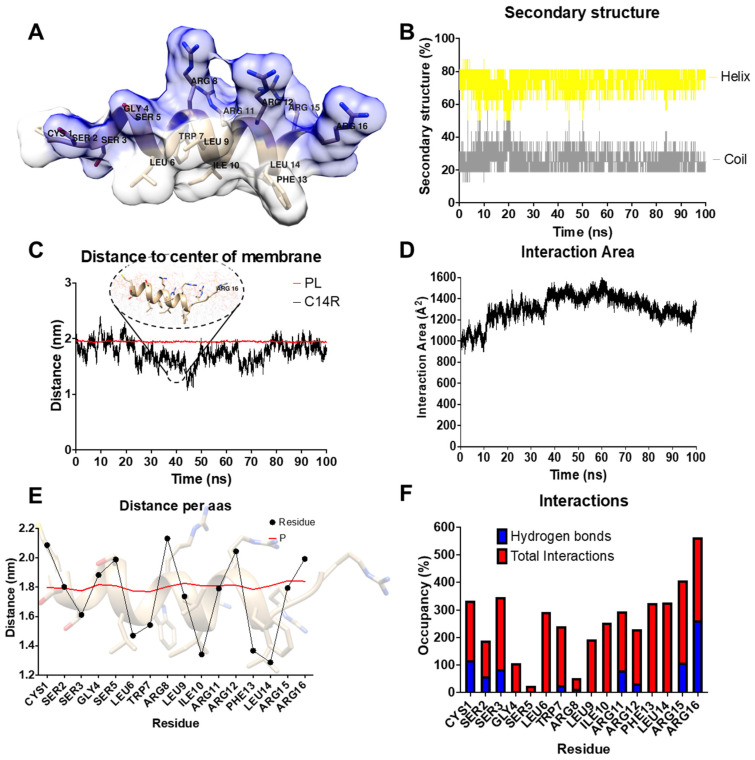
In silico study of the interaction of the peptide C14R with *P. aeruginosa.* (**A**) The 3D structure of the peptide C14R obtained by an ab initio method. The peptide is represented in tan ribbons and residues in licorice. The amphipathic character is represented in white and blue surface representations for polar and non-polar residues. (**B**) Percentage of secondary structure, helix (yellow) and coil (gray). (**C**) Distance of the peptide (black) and phospholipid heads (red) to the center of the membrane. (**D**) Area of interaction of the peptide with the bacterial membrane. (**E**) Distance to the center of the membrane per residue. (**F**) Total of interaction (red) and hydrogen bonds (blue) per residue in the last 10 ns of simulation.

In order to evaluate the possibility of membrane activity and, hence the resulting antimicrobial effect of the peptide C14R against *P. aeruginosa*, first a theoretical approach using molecular dynamics simulations was carried out. The 3D structure of C14R was predicted using a deep learning algorithm implemented in AlphaFold2 (Figure 1 and Figure 2A). Recent works have demonstrated that AlphaFold2 can accurately predict α-helical, β-hairpin, disulfide-rich, and cyclic peptides [27,28]. The 3D structure of the peptide obtained shows an α-helix conformation with a high amphipathic character, with the hydrophobic residues being located in one side (white surface) of the helix while the polar residues are in the opposite one (blue surface) (Figure 2A). The structure of the peptide shows high stability in its interaction with the membrane during the whole simulation, with a 75% helix and 25% coil structure (Figure 2B). The adsorption steps of the peptide to the bacterial membrane were analyzed by measuring the distance of the center of mass (COM) of the peptide to the COM of the membrane and phospholipid heads. The peptide showed a tight association with the membrane where it passed the phospholipids barriers at some points of the simulations (25–80 ns). However, the arginine 16 in the C-terminus established a strong interaction with the phospholipids head, avoiding the complete internalization of the peptide (Figure 2C). This result is in correspondence with the interaction area analysis, showing a higher interaction (1400 Å^2^) from 40 to 70 ns where the peptide is deeper inserted in the membrane (Figure 2D) before returning to the previous conformation. To determine the orientation of the peptide in the membrane, the distance of the COM of each residue to the membrane’s center was measured though the function of time and was related to the phospholipid heads. The peptide acquired a conformation parallel to the membrane’s surface during the whole simulation. It oriented the non-polar residues to the hydrophobic patch of the membrane composed off the lipid tails, while the polar residues such as arginine were placed in the aqueous environment (Figure 2E). This orientation allows for the peptide to interact with the membrane through almost all of its residues. In the literature, the described aromatic residues Trp, Tyr, and Phe are believed to act as anchors [29]. C14R has two aromatic residues, TRP7 and PHE13, both with more than 300% of occupancy during the last 10 ns of the simulations. This value indicates that these residues were interacting with more than one lipid at the same time. Additionally, the non-polar residues present a high number of interactions that are absent of hydrogen bonds, indicating hydrophobic interactions. On the other hand, polar residues such as arginine 11, 15, and 16 have a high number of both interactions, hydrophobic and hydrogen bonds (Figure 2F).

The results presented above point to the idea that the peptide C14R could exert antimicrobial activity against *P. aeruginosa.* The peptide adsorbs to the membrane, helped initially by electrostatic interactions facilitated by the arginine residues. Then, it is located to the aqueous–membrane interface, helped by its amphipathic character and the aromatic residues Trp and Phe, which can act as anchors. This interaction remains stable until the peptide reaches a critical concentration to form complexes, allowing it to penetrate the membrane and exert its mechanisms. However, further studies are required to approve or reject this hypothesis.

**Figure 3:**
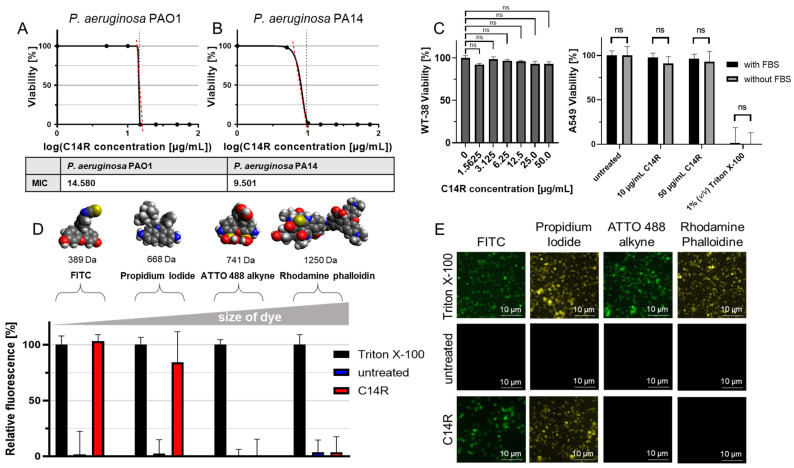
C14R anti-*P. aeruginosa* activity and pore-forming capability. (**A**) C14R MIC determination using increasing peptide concentrations with *P. aeruginosa* PAO1 and (**B**) PA14. Respective MICs (Table) were calculated using non-linear curve fitting with the Gompertz equation. Red dashed line represents the slope, grey dashed line represents the logMIC values. (**C**) Cytotoxicity analyses. Left panel: cytotoxicity was measured by an MTT viability assay using human lung fibroblasts WI-38. Right panel: cytotoxicity was measured by a resazurin reduction viability assay using adeno-carcinomic human alveolar basal epithelial cells A549 with or without FBS supplementation. Standard deviations represent experiments conducted as triplicate with significance testing using the Student’s *t*-test with the result “non-significant” (ns). (**D**) Permeabilization assay using *P. aeruginosa* PAO1 and treatment with 15 µg/mL C14R for 2 h. Staining of porous cells using FITC, propidium iodide (pI), ATTO 488 alkyne, or rhodamine phalloidin as fluorescent dyes with Triton X-100 as positive control agent. The experiments were conducted in triplicate. (**E**) Fluorescence microscopy analysis of samples from (**D**) using the Leica DMi8 (Leica Microsystems CMS GmbH, Wetzlar, Germany) at 630× magnification.

The theoretical ability to interact and insert into biomembranes (i.e., the inner membrane) of *P. aeruginsosa* was then verified experimentally by measuring the impact of increasing concentrations of the C14R peptide on the viability of the pathogen during planktonic growth in a standard microbiological medium. By plotting measured viabilities and C14R concentrations, a non-linear fit using the Gompertz equation for MIC determination delivered MICs of C14R for the reference strains *P. aeruginosa* PAO1 [30] and PA14 [31] of 15 and 10 µg/mL, respectively (Figure 3A,B). With their pore-forming ability, classical AMPs carry with them an intrinsic obvious drawback, since their activity in principle relies on a self-assembling process into the hydrophobic background of the biomembrane, thereby forming a hydrophilic channel in the pore complex [19,20,31]. This pore formation is not only toxic to pathogenic target cells as desired but can also be incompatible with the viability of human cells, a side-effect endowing those peptides with considerable cytotoxic activities. However, examples which meet a fruitful balance between effective killing of *P. aeruginosa* cells and sparing human cells have been described for AMPs, allowing for the highest levels of their viability and qualifying such molecules as non-cytotoxic AMPs. The cytotoxicity of C14R was analyzed using human WI-38 lung fibroblast cells as an exemplary cell line with concentrations of C14R up to 50 µg/mL and an exposition time of 24 h. According to the European EN ISO 10993-5 norm, cell viabilities above 70% are considered as non-toxic effects [32]. However, in the case of C14R, with all values being close to 100% viability, all peptide concatenations tested proved to have non-significant differences to the untreated negative control, thereby impactfully outranging the minimum standard of EN ISO 10993-5 (Figure 3C, left panel). Cytotoxicity was then analyzed using the adeno-carcinomic human alveolar basal epithelial cell line A549. To omit the possible side-effects of the supplementation of FBS in cell culture media, two independent assays were performed and again no toxic effects were determined in the analyses with and without FBS (Figure 3C, right panel). The surfactant Triton X-100 served as a positive control for cytotoxicity, strongly affecting A549 cells (Figure 3C, right panel). The overall non-toxicity was additionally supported by a hemolysis assay using Colombia blood agar (Figure S1) with no observable hemolytic activity for C14R and the control antimicrobial peptides LL-37 [33] and Cm-p5 [8,10]. The target bacteria themselves served as additional controls, causing significant hemolysis by their known hemolytic activity. To supply additional evidence and to prove the predicted pore-forming capability of C14R, a permeabilization assay was performed with living *P. aeruginosa* PAO1 cells as an example. These cells were confronted with C14R at the previously determined MIC of 15 µg/mL for *P. aeruginosa* PAO1 in the presence of four fluorescent dyes of different molecular sizes (FITC < propidium iodide < ATTO488 alkyne < rhodamine phalloidin), along with the detergent Triton X-100 as an ultimately pore-forming positive control agent (Figure 3D). Whereas FITC and propidium iodide could enter the cells unhampered, ATTO488 alkyne and rhodamine phalloidin remained perfectly excluded from entering the cells (Figure 3D). This demonstrates that C14R not only forms pores, but also suggests a defined cut-off size for molecules to enter the cytoplasm via this gate. As expected, Triton X-100 permeabilized *P. aeruginosa* PAO1 perfectly, with all dyes being internalized. Fluorescence microscopic analyses of C14R-treated cells and Triton X-100 in the presence of the dyes comprehensively confirmed the results of the fluorometric penetration assay (Figure 3E).

**Figure 4:**
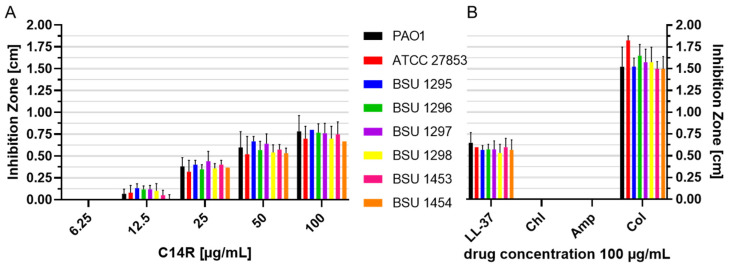
Overlay radial diffusion assay on trypticase soy agar. (**A**) The inhibition zones [cm] were determined with increasing concentrations of C14R peptide and (**B**) with LL-37 serving as control peptide, chloramphenicol (Chl) and ampicillin (Amp) as conventional antibiotics and colistin (Col) as peptide antibiotic for six clinical *P. aeruginosa* isolates (BSU 1295, 1296, 1297, 1298, 1453, and 1454) and *P. aeruginosa* PAO1 and *P. aeruginosa* Boston 41501 (ATCC 27853) as reference strains. Error bars represent standard deviations.

Intending to introduce a molecule as a lead structure for a potential new antibiotic drug, it was necessary to at least perform initial testing of C14R activity on *P. aeruginosa* strains of higher clinical importance than the laboratory reference strains. Thus, a preliminary set of six clinical *P. aeruginosa* isolates collected at the University Hospital Ulm were tested for susceptibility against different concentrations of C14R in a radial diffusion assay used as a standard diagnostic assay in clinical microbiology. *P. aeruginosa* PAO1 and, in addition, a clinical reference strain (*P. aeruginosa* Boston 41501 (ATCC 27853)) were used as controls. Also, in this assay concentrations above 10 µg/mL (i.e., starting from 12.5 µg/mL in Figure 4A) led to considerable growth inhibition in all strains. A dose-dependent effect could be observed, allowing for the estimation that C14R affected all strains with a very similar effectiveness (Figure 4A). The human cathelicidin LL-37 served as a reference peptide in this set of experiments at a concentration of 100 µg/mL, and this was also the highest concentration tested for C14R, showing at least slightly bigger inhibition zones than LL-37 (Figure 4B). In contrast, for the two conventional antibiotics chloramphenicol (Chl) and ampicillin (Amp) no inhibition zones were detectable at a concentration of 100 µg/mL (Figure 4B). However, colistin, which is used as a last-resort treatment for multidrug-resistant Gram-negative infections including pneumonia, unsurprisingly showed effectiveness against all tested strains.

## 3. Discussion

Synthetic AMPs can be designed based on the existing knowledge of sequences, chemical properties of amino acids, their distribution in the sequence and ultimately based on structural information about already described and well-characterized pharmaceutically active molecules [22,25,33]. Nature’s design concept behind such pore-forming peptides appears to be strikingly simple since it relies on the distribution of hydrophobic and hydrophilic side chains of the amino acids in the peptide sequence on opposite sides of the molecules, which often possess only a single α-helix as secondary structure element [34]. The C14R interaction with membrane surfaces involves several events, the first of which is the attraction of cationic AMP residues to negatively charged lipids in the bacterial target membrane through electrostatic forces [35]. This could be because, after the electrostatic interaction, it is necessary to reach a critical concentration of peptides to induce self-association and full or partial lipid bilayer penetration. Once situated in the membrane core, AMPs can exert their action through different mechanisms [35]. A well-known conserved pattern for AMPs is their amphipathic conformation due to the polarity and concentration of hydrophobic residues facing one side of the helix and polar residues residing on the other face. This feature is crucial for antimicrobial peptides’ activity, as the cationic polar domain is particularly important for the initial interaction with the membrane surface. In contrast, the hydrophobic patch will drive the peptide insertion into the hydrocarbon chain membrane core, a process mostly mediated by hydrophobic and van der Waals interactions [36]. However, complete insertion was not possible, maybe because after the electrostatic interaction it is necessary to reach a critical concentration of peptides to induce self-association and full or partial lipid bilayer penetration [35]. Nevertheless, from the experimental results it is possible to conclude that when this critical concentration is reached, multiple peptide molecules aggregate to form oligomeric structures within the lipid bilayer. The hydrophilic regions of the peptides line the interior of the pore, providing a water-filled channel that disrupts the barrier function of the lipid bilayer and allows for the passing of fluorophores. The formation of the pore disrupts the integrity of the cell membrane, which leads to leakage of cellular contents, loss of membrane potential, and ultimately cell death [37]. The designed 16 amino acid sequence of C14R suffices this simple blueprint, which was expected to have the (pharmaceutical) features of a properly functional AMP. Moreover, C14R contains an amino-terminal cysteine residue providing a single thiol-group to the molecule, which may facilitate possible beneficial chemical modifications including directed and site-specific dimerization for certain applications. Over the course of experiments performed to prove that C14R fulfills these expectations, molecular dynamics simulations revealed that the peptide in fact has the ability to penetrate a model membrane composed according to the lipid “recipe” of a typical *P. aeruginosa* cell (i.e., *P. aeruginosa* PAO1) [38]. To substantiate these more theoretically approved findings, and the second essential property, which lies in the capability to form peptide aggregates as amphipathic pore-like structures in the phospholipid bilayer of the target biomembrane, a permeabilization assay was performed. Although the fact that in the series of fluorescent dyes with increasing molecular weight, only the two smallest molecules were taken up upon C14R exposition, it can be suggested that the C14R-based pore possesses a molecular size cut-off in this range. This is currently unclear, and whether it is really only a size cut-off or a whether the chemical nature of the dyes may influence their uptake needs to be evaluated. Pharmaceutically more relevant and essential for future applications of C14R was the experimental affirmation that the peptide affected the viability of *P. aeruginosa* cultures in a dose-dependent fashion with deduced MIC values of 10 and 15 µg/mL for *P. aeruginosa* PA14 and PAO1. Interestingly, the MIC was lower for *P. aeruginosa* PA14, although this strain is regarded as drastically more virulent than PAO1 [39,40,41], implicating that decreased sensitivity (i.e., increased resistance) is not necessarily linked to the pathogenic potential of an individual strain. A clinically essential broader anti-*P. aeruginosa* activity of C14R was verified with an initial set of six clinical isolates collected from patients of the University Hospital Ulm in a radial diffusion assay in clinical microbiology. The assay was performed with AMP LL-37 as a reference and positive control. This peptide is generally regarded as a potent pore-forming AMP and has been the object of optimization by design strategies [33]. Remarkably, the results of this assay implicated that C14R may not only be of competitive efficiency but may even exceed the antimicrobial potential of LL-37 as a well-established prototype AMP after further optimization processes. Another promising feature of C14R is its non-hemolytic activity accompanied by its marginally low or absent cytotoxicity as determined with the lung fibroblast cell line WI-38 and the lung epithelial cell line A549, respectively, as a correlation between the hemolytic activity and cytotoxicity of synthetic antimicrobial peptides and in vivo systemic toxicity has been discussed [42]. Although C14R anti-*P. aeruginosa* activity has so far only been evaluated with an ensemble of rather limited diversity of clinical isolates, and the cytotoxicity testing was conducted only with a single human cell line, we consider the obtained results to be promising. Based on these findings, in depth characterization of C14R is justified in the future, which includes the testing of large ensembles of clinical isolates ideally containing multi-drug resistant strains of *P. aeruginosa* and other relevant bacterial pathogens. Another aim of such follow-up studies will be to improve the knowledge on C14R-dependent toxicity towards a series of different cell lines. However, our study may already qualify C14R as a potent lead structure in the development of new peptide-based antibiotics in the future.

## 4. Materials and Methods

### 4.1. Molecular Dynamics Simulations

First, the peptide properties were determined using the ProtParam analysis tool (ExPASy) according to the literature [43].

In order to explore the ability of the C14R peptide to interact with the *P. aeruginosa* membrane, a 100 ns molecular dynamic simulation was performed. The membrane was prepared using a lipid composition mimicking the *P. aeruginosa* one: dioleoylphosphatidylglycerol (DOPG), dioleoylphosphatidylethanolamine (DOPE), and tetramyristoylcardiolipin (TMCL2) in a ratio of 60:21:11 [38]. The membrane was generated using an input generator from the website CHARMM-GUI (https://www.charmm-gui.org (accessed on 22 May 2023)) [44,45,46,47]. On the other hand, the 3D coordinates of C14R were predicted by ab initio modeling using a deep learning algorithm implemented in AlphaFold2 from Google Colabs (https://colab.research.google.com/github/sokrypton/ColabFold/ (accessed on 21 May 2023)) [48]. A peptide molecule was added to one side of the membrane with its center of mass (COM) at 20 Å from the COM of the membrane, mimicking in vitro experiments in which peptide molecules are initially added to the bacteria culture. All simulations were performed using the NAMD 2.14 package [49] with the CHARMM36 force field [50,51,52]. The TIP3P water model was used to generate explicit solvation conditions [53], and Newton’s equations of motion were integrated using the Verlet (leapfrog) algorithm [54]. Periodic boundary conditions were applied in all directions, and the cutoff of short-range van der Waals interactions was 1.2 nm. The particle mesh Ewald method [55] was applied to treat long-range electrostatic interactions, with a 1.2 nm real-space contribution cutoff for Coulombic interactions. A temperature of 310 K° and a pressure of 1 atm were maintained by the Langevin thermostat [56] and barostat [57], respectively. In all systems, the protonation states of peptides were assigned based on calculations at pH 7 and with 150 mM NaCl. The systems were equilibrated in two steps. First, a 1000-step minimization followed by 0.5 ns of equilibration with the protein constraint was performed to guide the system to the nearest local energy minimum in configuration space. Secondly, the peptide was released from the harmonic constraints and the whole system was further equilibrated by another 0.5 ns. After the equilibration process, all simulations were performed for 100 ns under an isothermal–isobaric (NPT) ensemble without any restraints.

### 4.2. Peptide Synthesis

The C14R peptide NH_2_-CSSGSLWRLIRRFLRR was synthesized automatically on a 0.10 mmol scale using standard Fmoc solid-phase peptide synthesis techniques with a microwave synthesizer (CEM Corporation, Matthews, NC, USA). In brief, a resin preloaded with arginine was used, provided in the reactor, and washed with dimethylformamide (DMF). The Fmoc protecting group was removed with 20% (*v*/*v*) piperidine in DMF, initialized with microwaves, and followed by washing with DMF. An amount of five equivalents of amino acids were added to the reactor, and then five equivalents of HBTU 2-(1H-benzotriazol-1-yl)-1,1,3,3-tetramethyluronium-hexafluorophosphate) were dosed into the amino acid solution, followed by the addition of 10 mol equivalents of *N*,*N*-diisopropylethylamine (DIEA). The coupling reaction was performed with microwaves in a few minutes, and then the resin was washed in DMF. These steps were repeated for all amino acids in the sequence. The last step was the final deprotection. Once the synthesis was completed, the peptide was cleaved by treatment with 95% (*v*/*v*) trifluoracetic acid (TFA), 2.5% (*v*/*v*) triisopropylsilane (TIS), and 2.5% (*v*/*v*) H_2_O for 1 h. The peptide residue was precipitated and washed with cold diethyl ether (DEE) by centrifugation. The peptide precipitate was then allowed to dry under vacuum to remove residual ether, and the peptide was purified using reversed-phase preparative high-performance liquid chromatography (Waters Corporation, Milford, MA, USA) (Figure S2A) with an acetonitrile/water gradient under acidic conditions on a Phenomenex C18 Luna column (Phenomenex Inc., Torrance, CA, USA) (5 µm particle size, 100 Å pore size) of dimensions 250 × 21.2 mm. After purification, the peptide was lyophilized on a freeze-dryer (Labconco Corporation, Kansas City, MO, USA) for storage prior to use. The molecular mass of the purified peptide mass was verified by liquid chromatography–mass spectrometry analysis (LC-MS) (Waters Corporation, Milford, MA, USA) and analyzed by matrix-assisted laser desorption time-of-flight-mass spectrometry (MALDI-TOF) (Figure S2B).

### 4.3. Cultivation of Pseudomonas Species

For antimicrobial testing, *P. aeruginosa* PAO1 [58] and *P. aeruginosa* PA14 [30] were grown in 5 mL Müller–Hinton broth (Carl Roth GmbH (Karlsruhe, Germany)) liquid cultures for 16 h at 180 rpm at 37 °C.

### 4.4. Viability Testing and Quantification

For *P. aeruginosa* strains, the minimal inhibitory concentration (MIC) of C14R and the viability of the bacteria were determined similarly to the “Clinical and Laboratory Standards Institute” guidelines M27-A3 broth microdilution assay [59]. In short, 2.5 × 10^3^ bacterial cells were seeded in 200 µL Müller–Hinton broth in 96-well polystyrene microtiter plates (Sarstedt AG & Co. KG, Nümbrecht, Germany) and incubated at 37 °C with agitation at 900 rpm on an Eppendorf shaker. The effect of C14R on cell viability was tested in the presence of the peptides at different concentrations, and the cell viability was quantified by a resazurin reduction assay according to Patricia Bi Fai et al. [60]. The cells were incubated with 20 µL of 0.15 mg/mL resazurin solution for 2 h. During this time, the viable cells reduced resazurin to the fluorescent resorufin by the production of NADPH. Cell viability was then quantified by measuring the amount of produced resorufin by fluorescence measurements at an excitation wavelength of 535 nm and an emission of 595 nm using a Tecan Infinite F200 microplate reader (Tecan Group Ltd., Männedorf, Switzerland). The curve was fitted using GraphPad PRISM 8 (Graphpad Software, Inc.; Boston, MA, USA) by a nonlinear fit using the Gompertz equation for MIC determination.

### 4.5. Cytotoxicity Testing

Cell viability was then measured by using the 3-(4,5-dimethylthiazol-2-yl)-2,5-diphenyltetrazolium bromide (MTT) (Thermo Fisher Scientific, Waltham, MA, USA) assay [61]. Therefore, the cell line WI-38 (ATTC number CCL-75) of human fetal lung fibroblasts was cultured in Dulbecco’s modified Eagle’s medium containing 10% fetal bovine serum, 50 U/mL penicillin, and 50 mg/mL streptomycin (Gibco, Gaithersburg, MD, USA) and incubated under a 5% CO_2_ atmosphere at 37 °C. The WI-38 cells (5 × 10^4^ per well) were seeded in a 96-well plate for 24 h at 37 °C in the corresponding cell culture medium before peptide treatment. Cells were treated with various concentrations of peptides for 24 h at 37 °C, and untreated cells served as controls. After the treatment, 20 µL of 5 mg/mL MTT solution was added to each well, and the plate was incubated at 37 °C for 2 h. Then, 100 µL of dimethyl sulfoxide was added into each well and incubated for 10 min to dissolve the formazan precipitate. The cell viability was detected by measuring the absorbance at 560 nm and 630 nm using a Tecan Infinite F200 microplate reader (Tecan Group Ltd., Männedorf, Switzerland). The obtained absorbance value of the control group was considered to be 100% cell survival, and the experiment was performed in triplicate.

Cytotoxicity was also examined in a resazurin reduction assay using the adeno-carcinomic human alveolar basal epithelial cell line A549 [62]. In brief, 2 × 10^4^ cells per well of a 96-well plate were incubated in 200 µL DMEM with additives at 37 °C and 5% CO_2_. One set of cells contained fetal bovine serum (FBS), whereas the other set of cells lacked FBS as an additive. After removing the medium, 100 µL of fresh medium, 100 µL of peptide solution (0, 10 or 50 µg/mL), and a 1% (*w/v*) solution of Triton X-100 served as a negative control. After an incubation time of 24 h at 37 °C and 5% CO_2_, 20 µL of a resazurin solution (0.15 mg/mL) was added to each well and further incubated for 24 h at 37 °C and 5% CO_2_. Fluorescence measurement (excitation wavelength—535 nm, emission wavelength—595 nm) of the converted resorufin was then performed using a Tecan Infinite F200 microplate reader (Tecan Group Ltd., Männedorf, Switzerland). Untreated A549 cells served as growth controls and all experiments were conducted in triplicate.

### 4.6. C14R Permeabilization Assay

To demonstrate C14R’s pore-formation capability in the *P. aeruginosa* membrane, a permeabilization assay was performed. Therefore, 10^7^ *P. aeruginosa* PAO1 cells were incubated in 200 µL Müller–Hinton broth supplemented with 15 µg/mL C14R and incubated for 2 h at 37 °C. For the positive control, 100 µL of a 0.1% (*w/v*) solution of Triton X-100 was added 10 min prior to the end of the 2 h incubation time, and for the negative control, no agent was added (untreated). After incubation, the tubes were centrifuged at 11,000× *g* and the supernatant was discarded, then the cells were washed with 1× PBS, and after the addition of 5 µL each fluorescent dye (FITC, propidium iodide (pI), ATTO 488 alkyne, or rhodamine phalloidin) and 195 µL 1× PBS (final concentration of dye: 5 µL/mL) for 20 min, the cell suspension was centrifuged at 11,000× *g* for 2 min, respectively. The supernatant was discarded, and the cells were resuspended in a 4% (*w/v*) solution of paraformaldehyde and incubated for 10 min in order to fixate the cells. Subsequently, the bacterial cells were washed 3× with 1× PBS, resuspended in 200 µL 1× PBS, and transferred to a flat-bottomed polystyrene microtiter plate with 96 wells (Sarstedt AG and Co. KG, Nümbrecht, Germany). Fluorescence measurements were conducted at excitation wavelengths of 498 nm (FITC), 535 nm (pI), 500 nm (Atto488 alkyne), 540 nm (rhodamine phalloidin), and emissions of 517 nm (FITC), 617 nm (pI), 520 nm (Atto488 alkyne), and 565 nm (rhodamine phalloidin) with a Tecan SPARK microplate reader (Tecan Group Ltd., Männedorf, Switzerland). Additionally, microscopic analyses were performed at 630× magnification using a Leica DMi8 fluorescence microscope (Leica Microsystems CMS GmbH, Wetzlar, Germany).

### 4.7. Overlay Radial Diffusion Assay

The activity of C14R was additionally measured in an overlay radial diffusion assay [63,64]. In short, *P. aeruginosa* strains were grown overnight in lysogeny broth (LB-Miller). Overnight cultures were washed with 10 mM phosphate buffer, and O.D.600 nm was determined. Then, 2 × 10^7^ bacterial cells were seeded in still-liquid agarose (Sigma-Aldrich) and, subsequently, a plate was poured. The wells were cut into the agarose with sterile wide bore pipette tips ((Axygen—a corning brand) and filled with C14R in concentrations ranging from 6.25 µg/mL to 100 µg/mL. As controls, the antimicrobial peptide LL-37 [33] (AnaSpec, Fremont, CA, USA), the peptide antibiotic colistin (Sigma-Aldrich, St. Louis, MO, USA), and the conventional antibiotics chloramphenicol (Sigma-Aldrich, St. Louis, MO, USA) and ampicillin (Sigma-Aldrich, St. Louis, MO, USA) were tested at a concentration of 100 µg/mL. Following 3 h of aerobic incubation at 37 °C, an overlay with trypticase soy agar (Oxoid) was conducted. Inhibition zones surrounding the wells were measured after overnight incubation at 37 °C and 5% CO_2_. For active peptides, three to five biological replicates were performed, and inactive peptides were repeated twice.

Along with *P. aeruginosa*, PAO1 *P. aeruginosa* BSU 856 (ATCC 27853) was used as a reference strain to compare C14R activity with *P. aeruginosa* clinical isolates. The strains were collected in an anonymized format and some have previously been published [64].

### 4.8. Hemolysis Assay

To analyze the possible hemolytic activity of the AMP C14R, the peptide and two reference peptides (LL-37 [33] and Cm-p5 [8]) were diluted to 10 µg/mL and 50 µg/mL and, additionally, overnight cultures of *P. aeruginosa* PAO1 (positive), *P. aeruginosa* PA14 (positive), 20% Triton X-100 solution (positive), and PBS (negative) served as hemolysis positive and negative controls. In short, reservoirs were cut into Columbia blood agar plates containing 5% sheep blood (Thermo Fisher Scientific Inc., Schwerte, Germany), 3 µL of each sample was added, and then the agar plate was incubated for 24 h at 37 °C, respectively. After incubation, the hemolytic activity of the peptides and the controls was determined according to the hemolytic reaction zones.

## 5. Conclusions

The affinity of the peptide C14R to *P. aeruginosa* membranes and its intrinsic tendency to productively insert into membranes of such composition were analyzed by dynamic simulations and the biological impact on the viability of two different *P. aeruginosa* reference strains was demonstrated with MICs in the range of 10–15 µg/mL. C14R’s pore-forming capability was verified in a permeabilization assay based on the peptide-triggered uptake of fluorescent dyes into the bacterial cells. Finally, the peptide was used in radial diffusion assays, where six clinical *P. aeruginosa* isolates were clearly affected, thereby paving the way for further in-depth analyses of C14R as a promising new AMP drug in the future.

## Data Availability

Data is contained within the article and Supplementary Material.

## Supplementary Materials

The following supporting information can be downloaded at: https://www.mdpi.com/article/10.3390/ph17010083/s1, Figure S1: Hemolytic activity on Columbia blood agar plates containing 5% sheep blood of the AMP C14R and the two reference peptides LL-37 and Cm-p5. Peptide concentrations 10 µg/mL and 50 µg/mL were analyzed and picked cultures of P. aeruginosa PAO1 (positive), P. aeruginosa PA14 (positive), 20% Triton X-100 solution (positive) and 1xPBS (negative) served as hemolysis positive and negative controls. Black dashed circles indicate the spatial dimension of the respective P. aeruginosa colonies inside the inhibition zone.; Figure S2, C14R Peptide Characterization. A) RP-HPLC analysis of synthetic C14R shows a high dominant signal corresponding to the pure peptide. B) MALDI-TOF spectrum of C14R. The m/z signal (2006.812) closely matches the expected theoretical m/z value of 2007.397.
