# Supplementary material for: The Designed Pore-Forming Antimicrobial Peptide C14R Combines Excellent Activity against the Major Opportunistic Human Pathogen Pseudomonas aeruginosa with Low Cytotoxicity
Source: Pharmaceuticals (Basel). 2024 Jan 9;17(1):83. doi: 10.3390/ph17010083 (PMC10820675; doi:10.3390/ph17010083)
Supplement: Supplementary file 1 [file pharmaceuticals-17-00083-s001.zip › pharmaceuticals-2735161-supplementary.pdf]

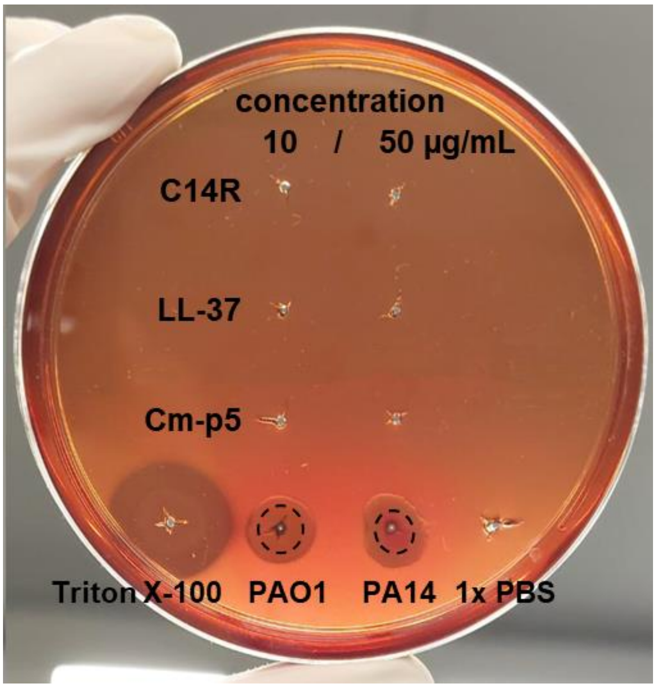

Figure S1. Hemolytic activity on Columbia blood agar plates containing 5 % sheep blood of the AMP C14R and the two reference peptides LL-37 and Cm-p5. Peptide concentrations 10 µg/mL and 50 µg/mL were analyzed and picked cultures of *P. aeruginosa* PAO1 (positive), *P. aeruginosa* PA14 (positive), 20 % Triton X-100 solution (positive) and 1xPBS (negative) served as hemolysis positive and negative controls. Black dashed circles indicate the spatial dimension of the respective *P. aeruginosa* colonies inside the inhibition zone.

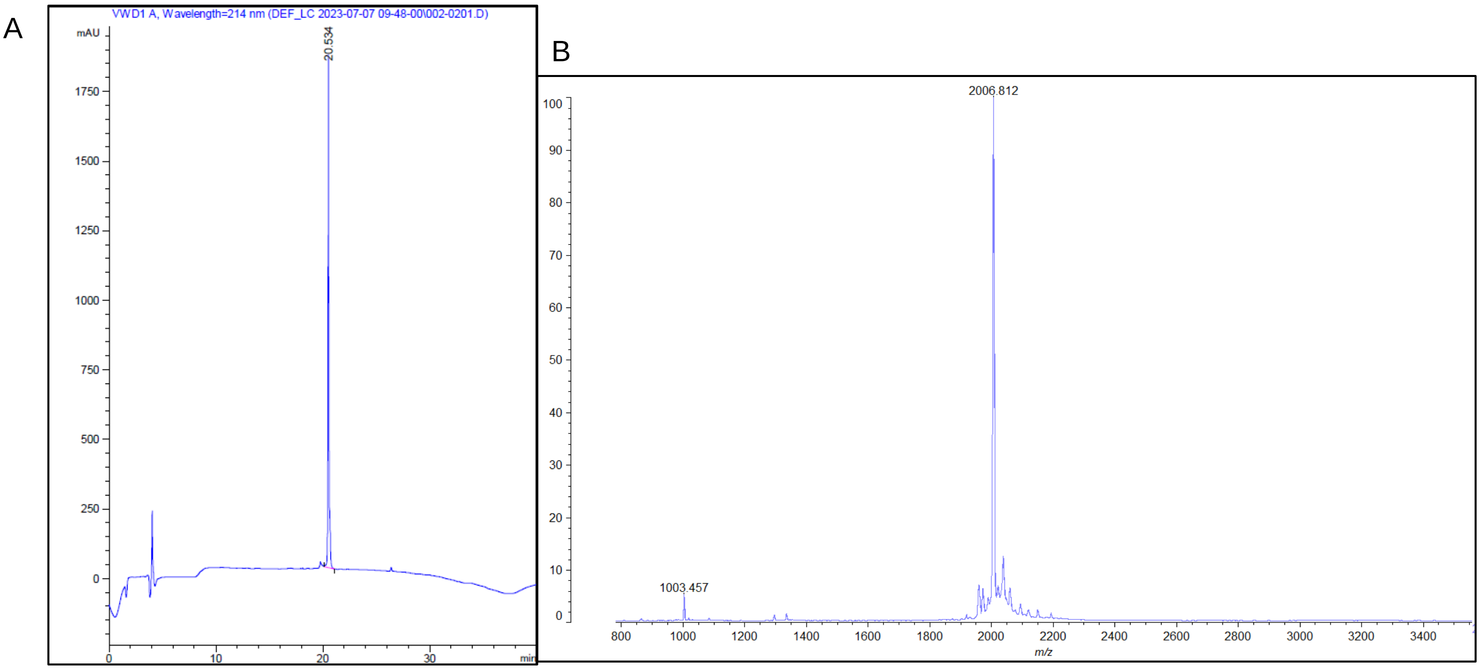

Figure S2. C14R Peptide Characterization. A) RP-HPLC analysis of synthetic C14R shows a high dominant signal corresponding to the pure peptide. B) MALDI-TOF spectrum of C14R. The m/z signal (2006.812) closely matches the expected theoretical m/z value of 2007.397.
